# Bilateral Calcaneus Transfers for the Treatment of Congenital Tibial Deficiencies: A Novel Surgical Technique and Case Report

**DOI:** 10.5435/JAAOSGlobal-D-22-00070

**Published:** 2022-12-05

**Authors:** William H. Fang, Evelyn Thomas, Juston Fan, Amber R. Leis, Samuel R. Rosenfeld

**Affiliations:** From the Orthopaedic Surgery, Riverside University Health System Medical Center, Moreno Valley, CA (Dr. Fang, Dr. Thomas, and Dr. Fan); the Orthopaedic Surgery, Children's Hospital of Orange County, Orange, CA (Dr. Fang, Dr. Thomas, Dr. Fan, Dr. Leis, and Dr. Rosenfeld); and the Plastics Surgery, University of California, Irvine, CA (Dr. Leis).

## Abstract

Tibial deficiency (also known as tibial hemimelia) is a rare condition with variable presentation. A 2-month-old patient presented with absent bilateral tibias. When the patient was 1 year, a novel reconstructive surgery was done. A bilateral fibular resection with pedicled calcaneus transfer was done, allowing for transfer of the calcaneus along with the overlying glabrous skin and soft tissues to the end of the femur. The patient was permitted to weight-bear after the 4-week postoperative follow-up. At the six-month follow-up, the patient was able to pull to stand and walk with assistance without any reports of pain.

Tibial deficiency (TD) is a congenital lower limb deficiency that involves a spectrum of deformities of the tibia, ranging from a hypoplastic to completely absent tibia. The fibula is present and can be normal or dysplastic. This condition is rare, presenting with an incidence of only one in 1 million live births, and is often subdivided into different types.^1^

There are many variations of TD, and commonly associated limb differences include absent patella and quadriceps mechanism, knee hyperextension or flexion, polydactyly, femoral deficiency, bifid femur, and foot disorders.^2–4^ Oftentimes, the condition presents itself as a shortened leg with knee and ankle deformities and cutaneous manifestations, such as dimples over the head of the fibula or at the end of the tibia.^5^ There are several different classification systems for the condition. The 1978 Jones classification is commonly used in clinical practice and separates the diagnosis into four presentations based on radiographic findings.^6^ In addition, the Weber^1^ classification, published in 2007, takes into account the entirety of the affected leg while further subdividing groups based on whether a cartilage anlage was present. Recently, the Paley classification was proposed in 2003,^7^ was modified in 2015, is based on the progressive deficiency and pathoanatomy of the presenting TD, and serves to guide surgical and reconstructive options.^5^

Owing to the rarity of TD and wide spectrum of presentation, there is no standard treatment or reconstructive procedure recommended. In many cases of TD presenting with tibial absence (Jones Type I or Paley Type 5), early amputation is suggested as treatment.^8^ However, isolated case reports discuss attempted reconstruction based on the amount of residual anatomy present. For example, in the Jones Type I presentation, fibular transfer or centralization, fusion, or arthroplasty have been reported with mixed outcomes.^9,10^ There are many advantages to retaining the foot, including better functional outcomes in most cases.^11^ This is likely because of the unique anatomic characteristics of the calcaneus and heel as weight-bearing tissues.

In this case report, we demonstrate the result of a novel limb salvage treatment of a young child presenting with bilateral tibial hypoplasia (Jones Type I, Paley type 5c). Informed consent was obtained from the mother for the publication of this case report.

## Case Presentation

A 2-month-old Hispanic male patient presented to our clinic with bilateral TD. He was born at 39 weeks' gestation to a 30-year-old GP4103 woman through cesarean section. His mother denied any history of illness, teratogen exposure, gestational diabetes, or complications throughout the pregnancy. She also denied any known genetic issues or birth defects in the family. During pregnancy, routine ultrasonography imaging indicated that the patient had absent bilateral tibias. Of note, the patient also had L5 left hemivertebrae, lumbosacral spinal dysraphism, and cryptorchidism. Management after his birth included bilateral inguinal orchiopexy and hernia repairs, which were uncomplicated. Genetic testing revealed loss of heterozygosity on microarray, and this was of uncertain clinical significance.

**Figure 1:**
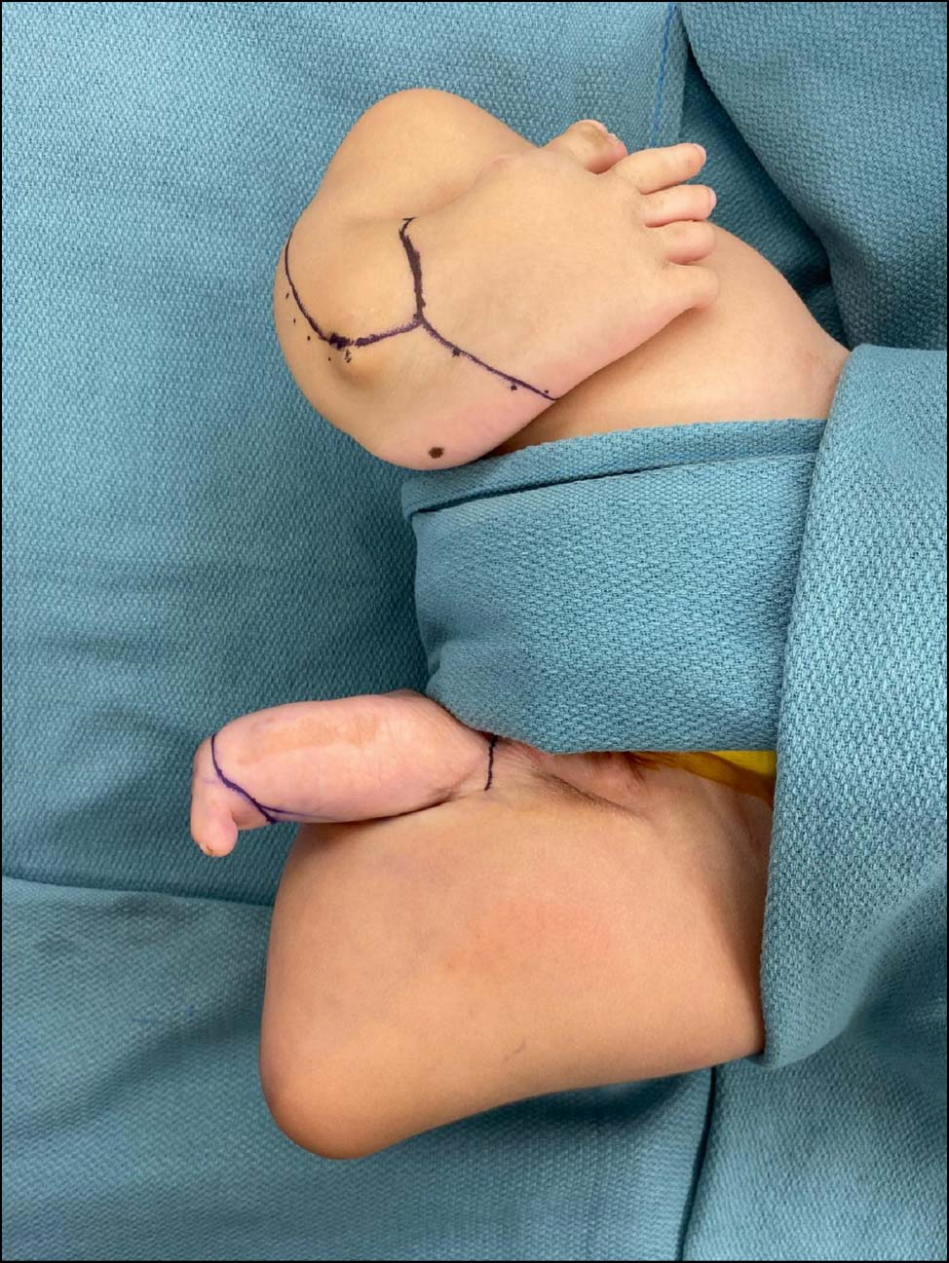
Preoperative image demonstrating shortened bilateral lower extremities with pterygiums and tibial deficiencies.

Clinical examination revealed bilateral popliteus pterygiums and shortened bilateral lower extremities with tibial deficiencies. Bilateral fibulas were present. Bilateral feet were noted to have rigid equinovarus and supination deformities (Figure 1). The left foot was hypoplastic with only one digit present. Dimpling at the knees was noted. The patient had full range of motion in bilateral hips, and the spine was palpably straight without a hairy tuft.

**Figure 2:**
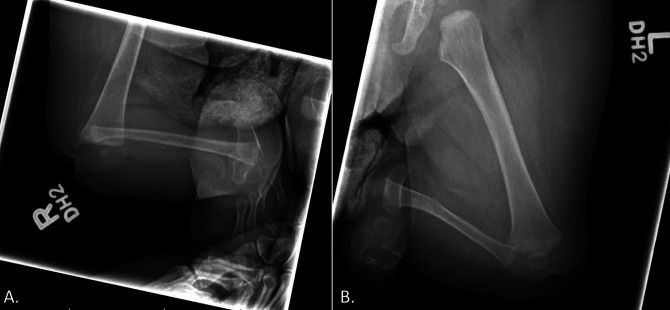
Radiographic anterior-posterior imaging when the patient was of 8 months. **A,** Right lower extremity demonstrating absent tibia with dislocation and severe flexion contracture of the knee and subluxation or dislocation of the ankle. **B,** Anterior-posterior view of the left lower extremity demonstrating absent tibia with dislocation or subluxation of the knee and dislocation of the ankle.

Initial plain radiographs of the right lower extremity demonstrated an absent tibia, with dislocation and severe flexion contracture of the knee and subluxation or dislocation of the ankle, Paley 5c classification (Jones I). Radiographs of the left lower extremity demonstrated the absence of the tibia and multiple bones of the foot on the left side. Subluxation or dislocation of the left knee and dislocation of the left ankle were also noted. A subtle deformity of the left distal fibular diaphysis with possible sclerosis was also noted (Figure 2). There was also an absence of the quadriceps and knee capsule on clinical examination. The decision to conduct bilateral pedicled calcaneus transfers around 12 months of life was made to preserve the weight-bearing structures of the lower limb and to allow for earlier weight bearing with prosthetics and earlier ambulation compared with other reconstructive procedures or primary amputation.^12^

## Treatment

We did surgical treatment of the patient at the age of 1 year and 24 days with a multidisciplinary team including orthopaedic surgery and plastic surgery. Careful surgical planning of incisions was done with flap designs according to the plastic surgeon. Throughout the procedure, the tissues were carefully dissected to allow for rearrangement, with the principles of flap length-to-width ratios of 2:1, and to minimize the need for healing of wounds by secondary intention. The flaps were designed to incorporate residual nerve structures to preserve sensation to the calcaneal skin and reduce the risk of neuroma formation. The calcaneus was preserved with the overlying skin used as a pedicled vascularized bone transfer. Owing to the complexity of the soft-tissue contractures and deficiencies, we allowed for the possibility of cosmetic contour revisions at a later date, rather than resecting tissues at the risk of vascular compromise.

**Figure 3:**
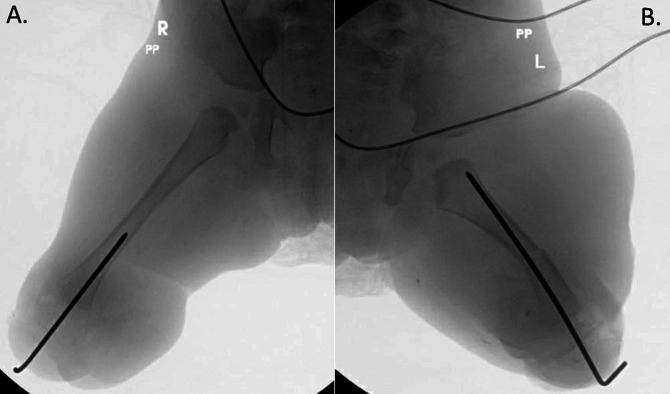
Intraoperative fluoroscopic anterior-posterior imaging of bilateral lower extremities. **A,** Right lower extremity demonstrating a smooth pin inserted through the calcaneus into the distal femur. **B,** Left lower extremity demonstrating a midshaft femur osteotomy with a smooth pin inserted through the calcaneus into the femur.

On the right side, the forefoot, midfoot, and talus were excised. The articular cartilage of the distal femur and calcaneus was also sharply excised as described by Boyd.^13^ The right fibula was carefully excised by subperiosteal dissection, preserving the vessels and nerves. Just enough cartilage was removed to achieve good apposition to facilitate arthrodesis. At this age, not all cartilages need to be removed for an arthrodesis because it will eventually turn into bone. The calcaneus, attached to the skin pedicle, was brought to the distal end of the denuded articular surface of the femur and fixated with a smooth Steinmann pin inserted through the plantar calcaneal skin, through the calcaneus, and into the medullary canal of the femur (Figure 3, A).

On the left side, the talus and calcaneus were in coalition; the talus was carefully dissected and removed from the calcaneus, as well as the remaining metatarsals and toes of the hypoplastic left foot. The left fibula was excised by subperiosteal dissection, preserving the vessels. During the removal of the fibula, the left peroneal nerve was sharply transected, and it was buried in muscles to prevent formation of a symptomatic neuroma. There was a severe knee flexion contracture, and the entirety of the left foot pad was tethered to the proximal groin with pterygium, which created a challenge for preservation of tissue vascularity during the transfer. The tethering structures included the deep muscle fascia of the femur, which was released. Femoral nerve neurolysis was done to allow for additional mobility. Although the tissues were mobilized maximally, the stretch required to reach the end of the femur resulted in blanching of the flap. To allow for a safe transfer of the calcaneus to the femur, the distal femur was shortened with midshaft osteotomy removing approximately 3 cm of the femur. A smooth Steinmann pin was then inserted through the heel pad and into the medullary canal of the femur as was done for the right side (Figure 3, B).

**Figure 4:**
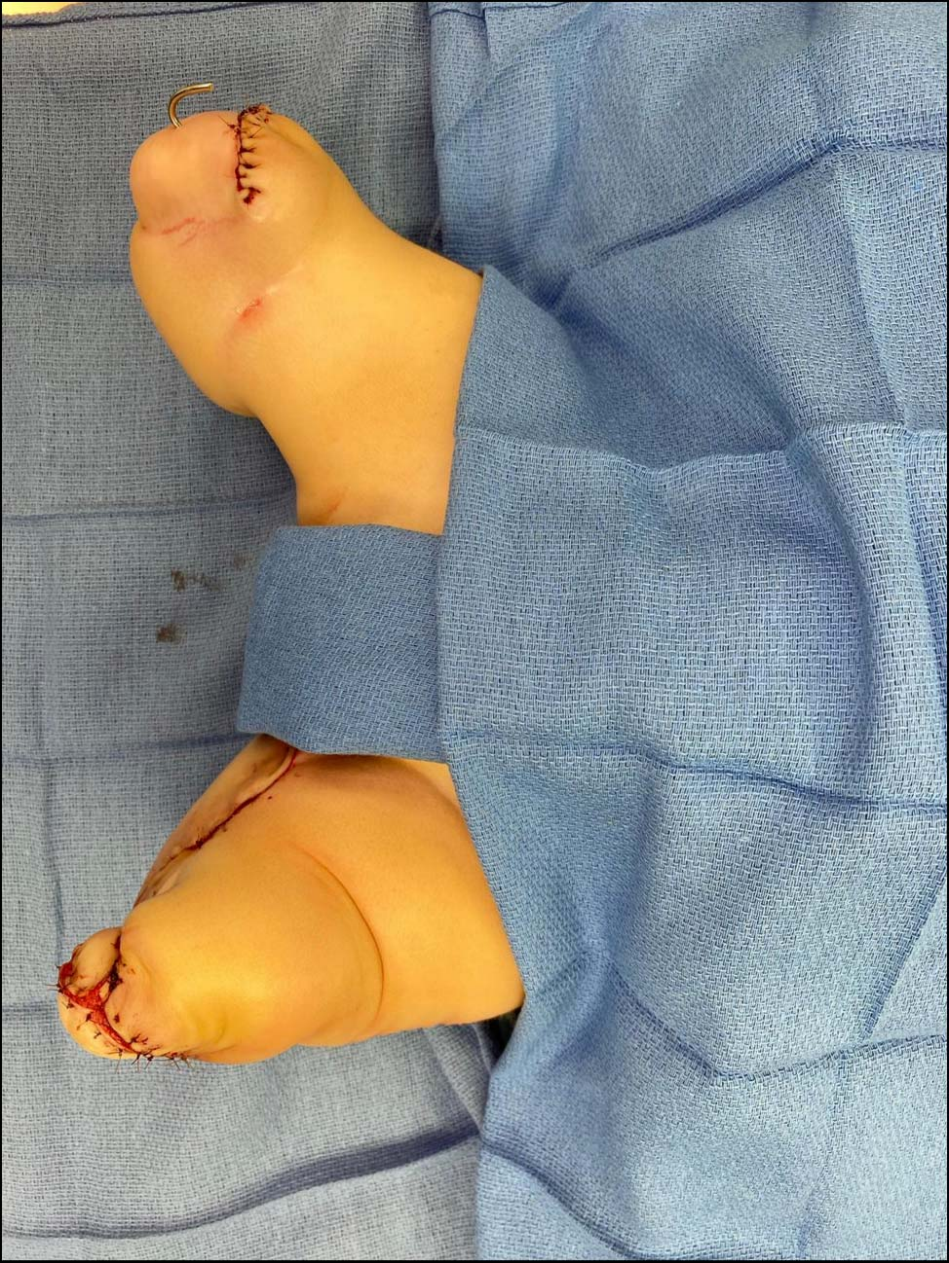
Postoperative image after bilateral calcaneal transfers and soft-tissue rearrangement.

The wounds were irrigated, closed, and dressed appropriately (Figure 4). Bilateral femoral nerve blocks were placed to decrease postoperative pain.

**Figure 5:**
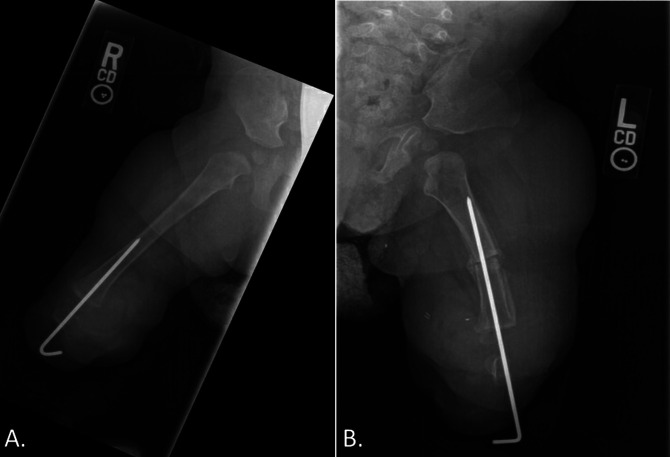
Radiographic anterior-posterior imaging of bilateral lower extremities at 1 month after procedure. **A,** Right lower extremity image demonstrating good alignment of the calcaneus and femur with bony healing. **B,** Left lower extremity image demonstrating a midshaft femur osteotomy with callus formation and appropriate alignment of the calcaneus and femur.

**Figure 6:**
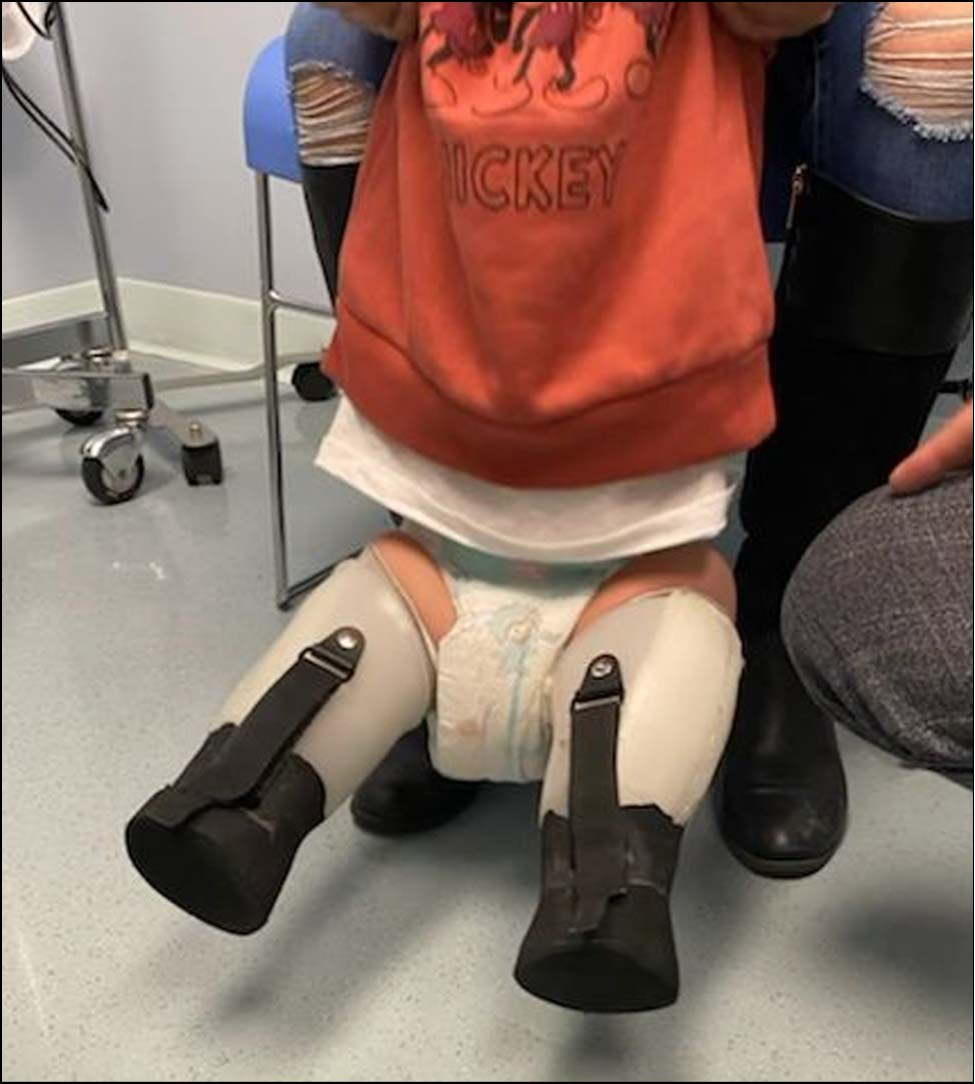
Photograph of the patient at the 4-week postoperative follow-up with prescribed Stubbies for weight-bearing activities.

The patient was kept non–weight-bearing for 4 weeks with soft dressings overlying the lower extremity. Dressings were changed in the clinic at regular intervals. The patient was permitted to bear weight after the 4-week postoperative follow-up. At that time, his wounds had clinically healed and appropriate bony healing of osteotomy and calcaneal transfer sites was demonstrated radiographically, allowing for Steinman pin removal (Figure 5). Stubbies were prescribed for weight bearing when outdoors (Figure 6).

**Figure 7:**
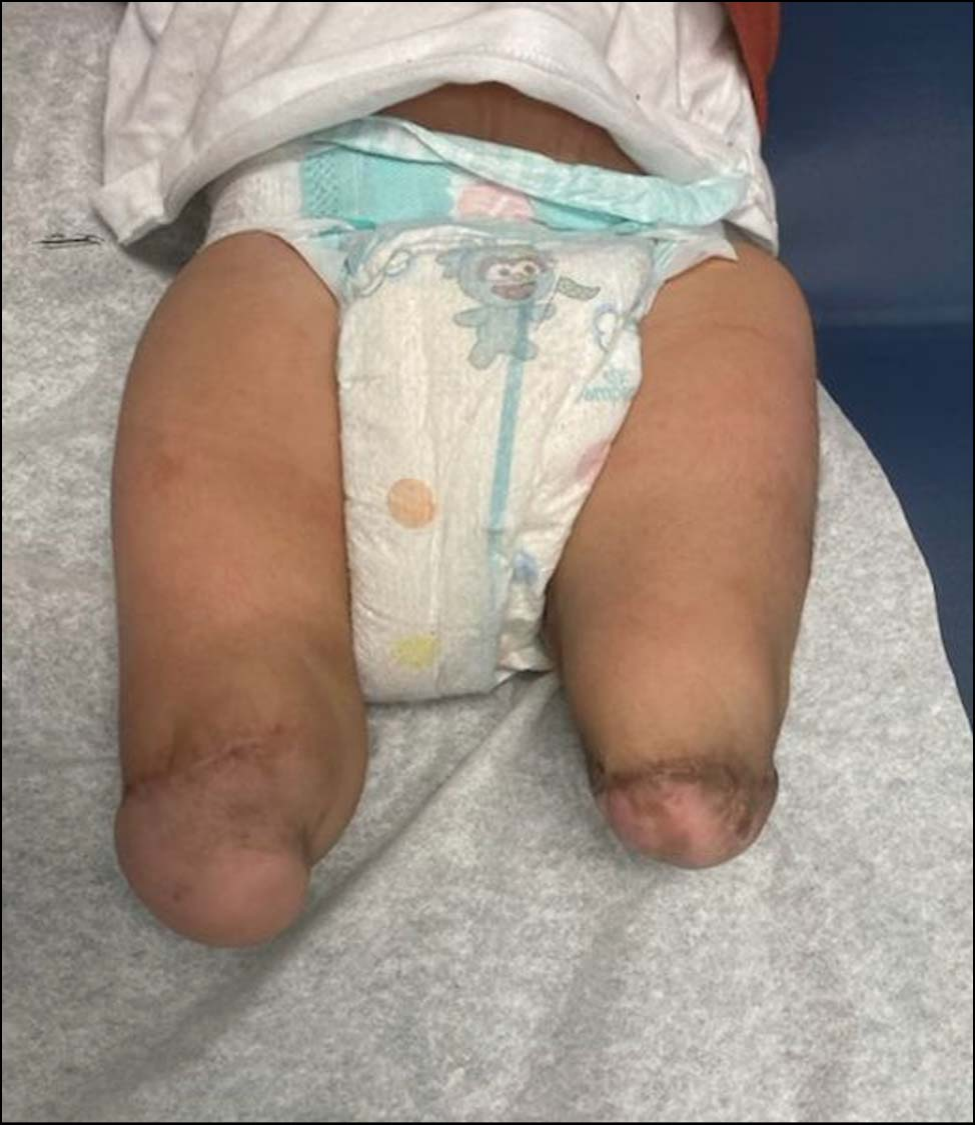
Photograph at the 8-week postoperative follow-up revealing adequate wound healing.

**Figure 8:**
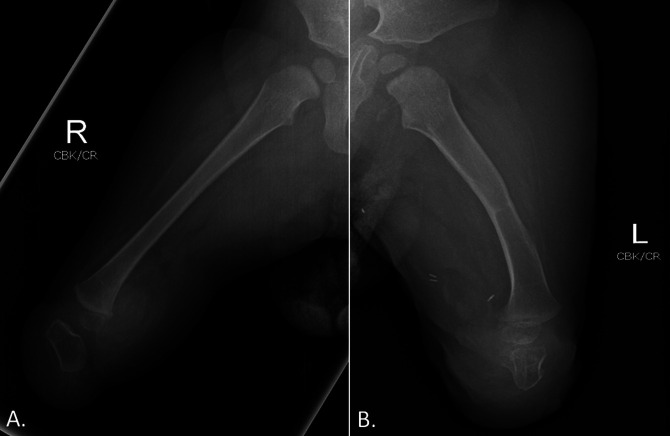
Radiographic anterior-posterior imaging of bilateral lower extremities at 9 months after the procedure, demonstrating cartilage-on-cartilage apposition of the distal femur and calcaneus. **A,** Right lower extremity demonstrating good alignment of the calcaneus and femur. **B,** Left lower extremity demonstrating healed midshaft femur osteotomy with appropriate alignment of the calcaneus and femur.

At the 8-week postoperative follow-up, the mother reported that the patient had been crawling without any signs or reports of pain and participating in all activities with adequate healing of the surgical site (Figure 7). At the 6-month follow-up, the patient was able to pull to stand and walk with assistance without any reports of pain. No complications have been noted up to date at the 9-month follow-up (Figure 8). In addition, Figure 8 demonstrates cartilage-on-cartilage apposition as described by Boyd.^13^ Radiograph evidence of arthrodesis will take years to visualize. The patient will continue to be followed routinely to monitor his progress.

## Discussion

Both the rarity and variety of presentations of TD make it a complex and challenging condition to treat. Many early attempts at reconstruction have reported poor outcomes with conversion to amputation. Some authors suggest that amputation is a more reliable treatment in more severe cases,^14^ with additional supporters claiming that early amputation leads to better functional outcomes and greater patient satisfaction than those who underwent reconstruction.^15,16^ However, advancements in surgical techniques have allowed better management and correction of deformities. A more recent study reported better outcomes with no notable functional or psychological difference between primary amputation or staged limb reconstruction, supporting that even more severe cases can be successfully treated with reconstructive procedures rather than amputation.^17^ In this study, a patient presented with bilateral tibial deficiencies, and bilateral pedicled calcaneus transfers were conducted to allow for earlier weight bearing compared with other reconstructive procedures. This procedure further offers the advantage of preservation of the glabrous weight-bearing tissues of the heel.

The classification of this patient's presentation was in line with Paley type 5c, with the complete absence of the tibia, absence of the patella, and dislocated fibula, or Jones type I, with the absence of a visible tibia.^18^ Previous reports of surgical management of this classification of TD involve using two temporary wires inserted at the fibula and the other hooked over the proximal epiphysis. Then, fixator rings were applied to reduce the fibula and knee contractures and repeated for distal ring and foot contractures.^19^ The goal was to align both ends of the fibula with the femur and tibia before a second-stage surgery, known as the Paley knee reconstruction, can be done.^20^ However, the primary issue with this procedure is that centralization of the knee and ankle to eliminate the contractures may take up to 5 to 6 months, delaying the time at which the patient is able to be ambulatory. The second-stage surgery involves femoral shortening, quadricepsplasty, transfer of the quadriceps muscle to the fibular head, and implantation of an internal articulated joint distractor.^2^ External fixators and wires are removed at 1 month and 6 months postoperatively, and the patient requires casting and a knee-ankle-foot orthosis.^2^ Long-term outcomes of this operation are unreported.

In the procedure we described, only a single surgery was required to achieve a functional outcome for the patient. A novel technique of bilateral pedicled vascularized calcaneus transfers was used. Although the left femur had to be shortened to avoid vascular compromise, the functional difference this shortening made is obviated by the child's growth potential. Postoperatively, the patient was only non–weight bearing for 4 weeks and then was prescribed Stubbies for outdoor weight bearing. This reconstructive procedure allowed normal developmental milestone achievement by avoiding prolonged immobilization. In addition to allowing for earlier weight bearing, this technique avoids multiple procedures and complex orthopaedic implants. We think that this novel method to treat TD might be considered in instances where simple amputation is refused, as an alternative to multistage reconstructive surgery, and when the advantage of earlier weight bearing and faster recovery postoperatively is desired. By working in a multidisciplinary manner with both an orthopaedic and plastic surgeon contributing to the care of this patient, we think we have described a novel treatment strategy with the potential to provide excellent outcomes when treating these challenging cases.
